# Relationships between Motivations for Food Choices and Consumption of Food Groups: A Prospective Cross-Sectional Survey in Manufacturing Workers in Brazil

**DOI:** 10.3390/nu12051490

**Published:** 2020-05-20

**Authors:** Anissa M. Souza, Ingrid W.L. Bezerra, Gabriela S. Pereira, Karina G. Torres, Raiane M. Costa, Antonio G. Oliveira

**Affiliations:** 1 Postgraduate Program in Health Sciences, Centro de Ciências da Saúde, Universidade Federal do Rio Grande do Norte, Natal, RN 59012-570, Brazil; anissasouza@hotmail.com (A.M.S.); gaabsp@gmail.com (G.S.P.); karinagomestorres@gmail.com (K.G.T.); raianemedeiros@hotmail.com (R.M.C.); 2 Nutrition Department, Centro de Ciências da Saúde, Universidade Federal do Rio Grande do Norte, Natal, RN 59078-970, Brazil; 3 Pharmacy Department, Centro de Ciências da Saúde, Universidade Federal do Rio Grande do Norte, Natal, RN 59012-570, Brazil; oliveira.amg@gmail.com

**Keywords:** food choices, food consumption, food preferences, food intake

## Abstract

Motivations for food choices may determine consumption, and understanding that relationship may help direct strategies for formulating diets. This study aimed to identify associations between motivations for food choices and consumption of food groups. An observational cross-sectional survey was conducted in 921 manufacturing workers from 33 companies in Brazil, based on a stratified two-stage probability sample. Motivations for food choices were assessed with the Food Choice Questionnaire, and intake of food groups was measured using 24-h dietary recall. Consumption was classified into 31 food groups defined according to their nutritional value and the NOVA classification. Data were analyzed with multilevel mixed-effects regression. The results showed that sensory appeal and price were the most important motivations for food choices, while ethical concern was less important. Sensory appeal was positively associated with consumption of industrialized condiments (*p* = 0.022), price showed a negative correlation with consumption of plant oils (*p* = 0.022), ethical concern showed positive correlation within consumption white meat (*p* = 0.065) and negative correlation within pasta dishes (*p* < 0.001). Regarding the NOVA classification, health correlated with an increase in consumption of unprocessed foods (*p* = 0.017) and weight control with a decrease in consumption of processed culinary ingredients (*p* = 0.057).

## 1. Introduction

The feeding phenomenon is a complex system that includes simultaneous interactions between various factors, dimensions and determinants that directly influence eating habits. Motivations for eating are influenced by preferences for consumption of specific food components, cultural background, social norms, economic factors, and physiological mechanisms, but also cognitive-affective, family, genetic, and epigenetic factors that affect personality characteristics and food choices [1,2]. Understanding these motivations can support strategies to improve the quality of food for individuals and groups.

This is important, especially in recent decades, when the prevalence of diet-related diseases, such as obesity, dyslipidemia, diabetes mellitus, and cardiovascular diseases (CVD) has grown globally [3,4]. The increase in the prevalence of obesity and noncommunicable diseases has been accompanied by a progressive food and nutritional transition, defined by the replacement of fresh and minimally processed foods with ultra-processed foods with high energy density and low nutritional quality. The unfavorable health effects of that change were enhanced by increased physical inactivity [5,6].

Among the changes seen in dietary motivations and eating patterns, research has shown that the increase in consumption of ultra-processed foods is directly associated with the high appreciation of sensory appeal, with the high intake of free sugars, of total, saturated and trans fats, and with low consumption of proteins, dietary fiber, vitamins, and minerals [7,8,9]. This food group has an obesogenic profile and a low amount of healthy components when compared to minimally processed foods and freshly prepared dishes and meals [10,11,12].

Therefore, it is important to examine what factors are associated with motivations for eating, and how understanding them can be useful in modifying those motivations. Effective strategies to stimulate healthy habits with significant positive effects, especially for modifiable behaviors such as consumption of unhealthy foods, include a complex understanding of the determinants of food choice [13,14,15].

An instrument that has been widely used in several countries to assess the relative importance of a number of factors related to individuals’ food choice [16,17,18,19,20,21] is the Food Choice Questionnaire (FCQ), originally developed by Steptoe et al. [1]. It is a questionnaire consisting of items measuring the importance of nine motivation dimensions: convenience, natural content, weight control, price, health, mood, sensory appeal, familiarity, and ethical concern.

Many different approaches to food consumption research have been undertaken to collect information about the association of diet-related illnesses with what was eaten [11,22,23,24], but without understanding why those foods were eaten. Other approaches looked into the motivations for eating, but specifically for the identification of dietary choices based on general cultural patterns of food choice [16,17,18,19,20,21]. Others investigated the association of food choices with sociodemographic and anthropometric characteristics [25]. The FCQ has also been used in some research to look at the reasons for consuming certain specific foods [13,26,27]. However, little is known about the correlation between food choice motivations and the consumption of all groups of food.

Our current research interests are on manufacturing workers, not just because they represent an important segment of the active population, due to its size and relevance to the economy, but also because of concerns emanating from global agencies, including the World Health Organization (WHO) and the International Labor Organization (ILO), who have been expressing widespread agreement that health, safety, and well-being of workers, who make up nearly half the global population, is of paramount importance [28]. In this context, it is generally accepted that an adequate, balanced and healthy diet will contribute to improving the health status and productivity of workers, a belief that has fueled a growing interest in using the workplace to promote healthy food choices [29].

However, some studies have shown that workers’ food choices are often nutritionally inadequate and that this can favor the emergence and/or worsening of chronic noncommunicable diseases [30]. Therefore, understanding eating behavior among workers and the reasons for ‘why they eat what they eat’ can help researchers and health professionals to develop food interventions in the workplace that are more efficient and adapted to different worker populations. The present study aimed to discover the motivations for food choices among manufacturing workers, and how these motivations are related to the food groups consumed.

## 2. Materials and Methods

### 2.1. Study Design

This study, conducted by the Nutrition Department of the Federal University of Rio Grande do Norte in Brazil between September 2017 and July 2018, was an observational, prospective, cross-sectional survey based on a probability sample of workers from manufacturing industries. Because of practical and logistic reasons, the survey was done in the State of Rio Grande do Norte, with a population of 3.5 million and located in Northeastern Brazil. All companies agreed to the research in writing. All subjects gave their informed consent for inclusion before they participated in the study. The study was conducted in accordance with the Declaration of Helsinki, and the protocol was approved by the Ethics Committee of the University Hospital Onofre Lopes (Project identification code 2.198.545/2017).

The sampling plan consisted of a combined, proportional stratified and multi-stage sample. The strata were sector of activity (food and beverages, non-metallic mineral, textile), company size (small, 20 to 99 workers; medium, 100 to 499 workers; large, more than 500 workers), and company refectory offering free lunch (no, yes). The study subjects were selected by two-stage sampling. The first stage consisted of a simple random sample of companies within each stratum, with sample size proportional to the total number of companies operating in the State in that stratum. The state’s Industry Federation (FIERN) provided the sampling frame consisting of all the companies operating in the state. The second stage was a simple random sample of workers within each company selected in the first stage based on a list of all the refectory users at lunch hour that was obtained from each company selected in the first stage.

Inclusion criteria were male and female workers over 18 years-old, who were company employees for more than one year, and users of the company refectory at lunchtime. Temporary workers, trainees, and pregnant women were excluded from the study.

Food choice motivations were measured with a validated Portuguese translation and transcultural adaptation to Brazil [31,32] of the Food Choice Questionnaire. This scale is composed of 36 items grouped into nine dimensions. Each FCQ item is introduced by the affirmative sentence ‘It is important to me that the food I eat on a typical day…’ and is answered on a four-point Likert scale ranging for ‘not important at all (1)’ to ‘very important (4)’. An unweighted average is calculated for each questionnaire factor, with higher scores indicating a higher perceived importance of that factor.

Before the fieldwork started, all interviewers in the research team were trained at the department’s nutritional assessment laboratory in height and weight measurement, following the procedures set out in the Brazilian guidelines for the collection and analysis of anthropometric data in health services [33], as well as in the application of the 24-h dietary recall (24HDR) questionnaires and the FCQ. After written approval was obtained from all participating companies, a company officer, usually from the human resources department, was contacted by phone for scheduling the survey visit and, at the same time, was asked to inform the workers about the scheduled visit and to prepare a roster of all company workers eligible for the survey. From that roster, a simple random sample of workers was obtained using a previously prepared randomization list created with a computer random number generator. Each company agreed to allocate a space (e.g., cafeteria, medical office) equipped with the necessary material to conduct the interviews. Data collection took place from Tuesday to Saturday at the time defined according to the company’s convenience, in the morning shift before the worker received the meal, or immediately before the afternoon shift. In this way, production was not affected by the survey, and the 24HDR would reflect the food consumed in a random working day. From one to four visits were done in each company, depending on the allotted time for data collection established by the company. Workers’ identifying information was not collected, and the anonymized data were subsequently entered into an Excel spreadsheet.

The following demographic and socioeconomic data were collected using a structured questionnaire administered by trained interviewers: age, sex, education, marital status, monthly income, and whether the worker attended some form of in-house formal professional training. Weight and height were measured to calculate the body mass index (BMI) with, respectively, a digital scale (Inner Scan, Tanita Corp., Tokyo) and a body height meter (Sanny, São Bernardo do Campo, SP, Brazil). Nutrition status was classified according to the World Health Organization (WHO) recommendations [34].

### 2.2. Classification of Food Groups

Data on food intake were collected based on 24HDR administered by trained and experienced interviewers. The workers were instructed to indicate all food and beverages (except water) consumed in a 24-h period, including preparation, appetizers, cooking methods, amount, seasonings, brand names, time, and place of the meal (in the workplace, at home or outside the home). The data collection was conducted from Tuesday to Saturday to ensure that the description of all consumption was equivalent to a typical day dietary intake.

The 24HDR model used was structured in the five-pass method developed by the U.S. Department of Agriculture [35] to enhance complete and accurate food recall and reduce bias in the collection of food intakes.

After dietary data collection, all the described food and beverage amounts were converted from portions sizes to grams and milliliters based on previously established criteria (direct weighing, photographic records, or food package labels), according to a model adapted from the ISA capital questionnaire [36,37]. Dietary energy consumptions were estimated based on the Brazilian food composition table [38], complemented when necessary by other food composition tables [39,40].

All the foods, preparation, and beverages reported in the 24HDR were categorized according to their food groups and processing method. Therefore, each food was assigned to one of four NOVA classes based on the nature, extent, and purpose of the industrial processing. NOVA classifies foods into four groups: Group 1, unprocessed or minimally processed foods; Group 2, processed culinary ingredients; Group 3, processed foods, and Group 4, ultra-processed foods [41,42,43].

Furthermore, 44 food groups were created according to the nutritional value and the food origin, following the food-based dietary guidelines [44,45], and each reported food was assigned to one of those food groups. Therefore, all types of fruits were combined into the fruits group, not including fruit juice because of the difference in composition. Red meat, white meat, and processed meat were organized into different groups, as well as vegetables and leafy vegetables. Brown, black, and green beans were all assigned to the beans group because they are commonly eaten together with rice at lunch, while legumes such as soybeans, lentils, and chickpeas were included in the other legumes group because they are consumed in diverse proportions and preparations.

The whole grains group included all products and food items described as ‘whole,’ including oats and oats-based foods, brown rice, biscuits, pasta, and bread wholemeal. We also separated margarine and plant oil into different groups due to the difference in the lipid profile of these sources of fat. Regarding added salt and sugar, we opted for categorizing them into exclusive groups, which allowed quantification of the amount consumed and the association of these culinary ingredients with the food choices dimensions. Information regarding salt and artificial sweeteners were described in grams, as they do not contribute to caloric content, but were presented as items commonly used in meal preparation or daily use.

Most freshly-prepared foods, which include items from several food groups, were disaggregated into their ingredients. A small number of freshly-prepared mixed foods that are mainly based on unprocessed and/or minimally processed foods, and are typical of the Brazilian culinary diet, were not decomposed and were classified in the food group with the highest contributing ingredient, and into Group 1 of NOVA classification. For example, a typical Brazilian preparation named ‘baião de dois,’ made with a mixture of rice and beans, which has proportionally more rice than beans according to traditional cooking recipes was classified into the rice group and Group 1. On the other hand, the typical Brazilian recipes ‘Pamonha’, ‘Canjica,’ and ‘Mungunzá,’ which are corn-based preparations made with other culinary ingredients such as sugar, coconut milk, and natural spices, were classified into the corn group and Group 1.

The classification of all food items reported in the 24HDRs into the 44 food groups was conducted by four nutritionists involved in the research with experience in food and nutrient consumption quantification. All classifications were double-checked by another two nutritionists and, whenever discrepancies arose in the classification, they were discussed until a consensus was reached among all researchers. The daily contribution of each food group to the total energy intake was calculated based on nutritional information according to food composition and expressed as a percentage of total daily kcal consumption.

### 2.3. Statistical Analysis

Formal methods for the calculation of sample sizes when multilevel models are used in the analysis of complex survey plans have not yet been developed, and sample size calculations are commonly based on suggested guidelines. One often-used method recommends that 30 clusters, with a cluster size of 30, are probably adequate to obtain unbiased estimates of effect sizes [46]. Accordingly, we defined a sample size of 30 companies with 30 workers from each company. To compensate for losses, we increased the level-2 and level-1 sample sizes by 10%, reaching a target sample size of 33 companies and 990 workers.

Statistical analysis was done with Stata 15 (Stata Corp., College Station, TX, USA). The estimates of population means and proportions considered the stratification by activity sector, company size, and refectory, as well as the two-level sampling, with the computation of sampling weights for each level based on information on the number of workers in every company that was provided by the State’s Industry Federation.

To investigate the association of each dimension of the FCQ with the food groups, a multilevel mixed-effects linear regression with an unstructured covariance structure was used. To test the association of each factor of the FCQ with each food group, the dependent variable was the percent contribution of the food group to total daily caloric consumption, and the independent variable was the score of that factor of the FCQ. The economic sector, company size, and refectory in the premises are crossed factors, and their combination defined 18 strata that were included in the model as fixed factors. Company and workers within companies are nested factors and were entered as random factors. Probability weights for companies and for workers conditionally on the selection of their company were used. Finite population corrections for companies and for workers were computed from data provided by FIERN. The same statistical method was applied to the analysis of the association of food groups with NOVA classes.

The *p*-values from the above analyses were corrected for multiple testing with the Hochberg procedure, and all reported *p*-values are two-tailed and multiplicity adjusted. As this study has an exploratory nature, in order to avoid missing relevant associations, the false discovery rate was set at 10%. In addition, in order to decrease the number of statistical tests, the 44 food groups were reduced to only those food groups that were consumed by at least 5% of workers in the previous day, based on the data collected in the survey from the 24HDR. Study data including survey weights are available in the Supplementary Data File.

## 3. Results

The study included 33 companies, of which 6 were large-sized, 14 medium-sized, and 13 small-sized. The food and beverage sector was represented by 14 companies, the non-metallic minerals sector by 6, and the textile sector by 13 companies. Seventeen companies offered lunch in a refectory. The median number of workers included in each company was 24 (range 19–59).

A total of 921 manufacturing workers were included, 383 from the food and beverages sector, 177 from the non-metallic minerals sector, and 361 from the textile sector. The sample average age was 38.3 ± 17.7 years, with 406 (44.1%) females. The population estimates of the demographic characteristics of manufacturing workers, based on the sampling plan and survey weights, is shown in Table 1.

Table 2 shows the mean values of each dimension of the FCQ. The dimensions that scored higher, revealing the greatest influence of those factors on the worker’s food choices were sensory appeal, price, and convenience, while ethical concern, mood, and natural content were the least important motivation for food choice.

A total of 517 foods, preparations, and beverages were reported in the 24HDR and were organized into the predefined food groups. Table 3 presents the food groups retained for analysis, with a description of the components of each group, the percentage of workers who consumed each group the previous day according to the 24HDR data, and the average contribution of each group in percentage of the worker’s total daily energy consumption. The combination of the rice and beans groups, which is the basis of the Brazilian food, accounts for the highest percentage contribution to total energy intake (approximately 13%), followed by white meat (9.42%), breads (8.59%), and red meat (7.77%). The food groups most often consumed by workers were coffee and vegetables (88.0% both), beans (84.4%), and rice (826%). Food groups as salt (99.8%), natural spices (94.5%), sugar (93.2%), and plant oils (86.8%), which are important culinary preparations constituents, were also consumed by a large fraction of the population.

Table 4 presents, for each of the four NOVA classes, the average energy consumption, the average relative contribution to the total daily energy intake, and the percentage of workers consuming food from that class. Most dietary energy intake came from unprocessed or minimally processed foods (55.6%), which agrees with the results presented in Table 3. Processed culinary ingredients contributed 8.03% to total dietary energy, processed foods 16.1%, and ultra-processed foods 20.6%. All four food groups defined by NOVA classification were consumed by all or nearly all the population.

The results of the analyses of the association of FCQ dimensions with the food groups are presented in Table 5. In view of these results and considering the dimensions that are more and less relevant for workers, we highlight the association between the health dimension and increased contribution to total daily energy intake of whole grains (0.72%., *p* < 0.001), leafy vegetables (0.16%, *p* = 0.053), and eggs (0.26%, *p* = 0.065) food groups, between sensory appeal and increased industrialized condiments consumption (0.09%, *p* = 0.022), between the weight control dimension and increased consumption of whole grains (0.83%, *p* = 0.003), tuber and roots (1.58%, *p* = 0.22), and fruits (0.62%, *p* < 0.001), and between ethical concern motivation and white meat (1.83%, *p* = 0.065).

On the other hand, there were negative associations between the health dimension and consumption of breads (−1.21%, *p* = 0.030) and pasta (−1.46%, *p* = 0.002), between sensory appeal and the eggs group (−1.61%, *p* = 0.009), between weight control and breads (−1.16%, *p* < 0.001), pasta (−1.53%, *p* < 0.001), and margarine groups (−0.22%, *p* = 0.011) and between ethical concern and the pasta group (−0.73%, *p* < 0.001).

The only associations identified between FCQ dimensions and NOVA classes were an increase of 3.07 percentage points (95% CI 1.34 to 4.79, *p* (multiplicity adjusted) = 0.017) in the consumption of unprocessed foods class for each 1 point increase in the health dimension, and a decrease of 0.34 percentage points (95% CI 0.56 to 0.11, *p* (multiplicity adjusted) = 0.057) in the consumption of the processed culinary ingredients class for each 1 point increase in the weight dimension.

Analysis by multilevel mixed-effects linear regression with economic sector, company size, and refectory in the premises as fixed factors, and company and workers within company as random factors. Statistical significance was set at the 10% level.

## 4. Discussion

It is known that consumer attitudes are mediated by several factors that, together, result in choices that determine the consumption pattern of each individual and interact with the cultural and environmental environment in which the individual is inserted [2]. Actually, a number of studies have investigated how social context, culture, and individual differences influence the motivations for choosing food. However, it is equally important to understand the relationships between the motivations of food choices and the food actually consumed [47], but such relationships have not been sufficiently investigated. The present study was the first to examine the relationships between the motivations of food choices and the consumption of different food groups. Because of its social and economic importance, the study focused in the population of workers in the manufacturing industry.

One of the findings of this study was that sensory appeal and food price were considered the most important factors in the daily choice of food, suggesting that the appearance, smell, taste, and cost of the food are the main motivations of food choice in manufacturing workers. On the other hand, ethical concern did not emerge as an important factor in motivating food choices.

There is a vast literature that corroborates these findings. In studies conducted to identify motivations of food choices across different countries and potential cross-cultural interactions, sensory appeal and price were the most important motivations reported of food choices, while the least important were ethical concern and familiarity [48]. In fact, several surveys using the FCQ that identified the motivations of food choices among European consumers and compared the perceived importance of these motivations between different countries, concluded that they gave high scores to sensory appeal and price, but familiarity and ethical concern received low scores [16,17,18]. Similarly, a recent study conducted in Brazil, that applied FCQ for validation and cross-cultural adaptation, identified a greater frequency of ‘very important’ responses in items of the sensory appeal dimension and greater frequency of responses ‘it is not important’ in the items of the ethical concern dimension [32].

However, other studies that have looked into geographical differences, such as a research conducted by Prescott et al. [49] that aimed to examine the differences in the motivations of food choices among Asian consumers, have shown a more evident cross-cultural difference. They concluded that only New Zealand consumers value sensory appeal highly, while concern for price was the most important dimension only in Japanese consumers. In turn, familiarity and ethical concern were classified as the least important motivations for food choices for Asian consumers.

On the other hand, the results of our study indicate that the ethical dimension was the least relevant for workers. The ethical concern motivation includes three items that involve concern for the country of origin of the food, the labelling, and the ecological packaging, but these items may not reflect all the complexity of this dimension. In addition, ‘ethical consumerism’ is an ambiguous term that means different things to different people, but it is a concept that covers important aspects, such as issues of animal welfare, human rights, country of origin, fair trade, health and anti-globalization, in addition to addressing environmental issues, concern and sustainable food production [50,51,52]. Thus, it is possible that the specificity of the issues of ethical values considered in the FCQ will also be responsible for the lower appreciation of motivation.

The present study identified positive and negative correlations between the motivations of food choice and the consumed amount of specific food groups. The correlations estimate the variation in the relative contribution of each food group to the total daily energy consumption, according to the different motivations of food choice. Therefore, the results showed that the greater the valuation of sensory appeal, the greater the contribution of industrialized condiments to the daily caloric intake. These items are flavor enhancers that give sensory properties to foods and are represented by industrialized sauces, salt-based condiments that in their composition contain chemical additives, normally used in culinary preparations to intensify the taste, smell, and appearance of food, which may explain the preference for these products. The only negative correlation associated with sensory appeal was the consumption of eggs, presumably because it is related to the lack of appreciation of the smell and texture characteristic of this food.

As noted earlier, the second most important motivation of food choices was concern about price. The finding of a negative association with plant oils, that is, the contribution of plant oils to total energy consumption decreases when concern about food prices increases, was surprising. A possible explanation is that plant oil is used locally in the preparation of meat and fish, which are pricy foods, and this could have brought about this negative correlation.

Another motivation of food choices that was valued by manufacturing workers was convenience. Regarding this dimension, we expected to identify a greater consumption of items associated with practicality, speed in preparation, and consumption of food, but our findings contradicted this. We observed that the higher the score for this dimension, the greater the energy contribution of red meat to the daily energy intake. Interestingly, the FCQ items in this dimension with the greatest frequency of ‘very important’ responses referred to access and availability to purchase food (‘Found in stores close to where I live or work’ and ‘It is easily available in stores and supermarkets’), while the item with the most frequent response ‘not important’ is related to the time of preparation of the food (‘It does not take long to prepare’). These findings indicate, in particular, that more important than the practicality of food consumption and preparation is the possibility of having easy access and better acquisition of food.

The health and weight control dimensions of food choices, known to be strong motivators of dietary behavior, showed important positive correlations. The results showed that higher scores in these dimensions were associated with higher consumption of foods perceived as healthy and unprocessed (according to the NOVA classification), such as vegetables, fruits, whole grains, tubers, and roots. This pattern confirms that these items correspond to the most frequently offered definition of healthy eating. A similar result was described in a study conducted with adults living in the United States for more than 10 years, using the short version of The Eating Motivation Survey (TEMS), which evaluated the association between food choices and composition of the most recent meal. Phan and Chambers [53] observed that people were more likely to choose vegetables, dairy products, eggs, fruits, and poultry products at various times of the day, especially when these respondents rated higher the dimensions of weight control and health.

It is interesting to note that the growing public interest in the benefits of healthy eating supports one of the techniques of social influence commonly used in public health campaigns: communicating rules or behavioral patterns that guide people about what they should eat or choose to eat. Health messages can be an important conditioning factor in the selection of healthier food choices and, therefore, it is easy to find rules related to food that are generally associated with important recommendations to prevent diseases and promote quality of life [54]. From this point of view, whole products and unprocessed foods are widely accepted as healthy eating and are, therefore, more often selected since people choose them because of health concerns and weight control.

On the other hand, the motivations health and weight control, as well as the motivations natural content and mood, presented similar negative correlations. Natural content is a dimension that is normally associated with health and weight control, and the similar negative correlations occurs because people concerned with health and weight control are also concerned with the composition of food and prefer not to eat products rich in artificial ingredients and energy-dense foods [2].

In turn, the influence of mood on eating behavior is a very complex relationship, involving factors such as hunger, satiety, and physiological reward mechanisms, expectations based on previous experiences, emotional coping mechanisms, and individual eating trends [55]. For this reason, well-being considers important aspects about health and mood, suggesting that health is seen as bodily and mental well-being [20].

Thus, the relationship between eating behavior that involves well-being, and messages of concern for health, which reinforce recommendations on healthy eating and healthy food choices, may explain the non-preference for specific food groups. With this in mind, the results indicate that higher scores in these dimensions are associated with lower consumption of items perceived as ‘unhealthy’ and that could interfere with ‘well-being,’ such as bread, pasta, margarine and salt.

In addition, our results point to a negative correlation between concern with weight control and processed ingredients for culinary use, indicating a reduction in the contribution of items such as sugar, oils and salt to the total daily energy intake.

Familiarity is a dimension of food choices closely linked to food that includes the items ‘is what I usually eat,’ is familiar to me’ and ‘is like the food I ate as a child’. As expected, the consumption of traditional and ‘familiar’ foods, such as beans and soups, are positively associated with this motivation. The results consolidate the idea that the preparation of these traditional dishes, based on unprocessed or minimally processed foods, is usually related to family recipes that are prepared in domestic kitchens and with the use of regional culinary ingredients. In addition, it reinforces that people eat among family and friends and positively value the memory and affective experience that involve food.

Regarding the least important motivation for food choices, the ethical concern, our findings show that the percentage of people who are concerned with ethical issues have a higher consumption of white meat and a lower consumption of pasta. This behavior shows consistency with the fact that people who are more sensitive to ethics in food production and in sustainability issues avoid or refuse the consumption of red meat due to the environmental impact caused throughout production process, preferring white meats. On the other hand, we have no clear explanation for the lower consumption of foods from the pasta group among workers that scored higher on the ethical concern motivation, and an explanation based on the literature could not be found.

In general, it was observed that the differences in the appreciation of these factors and dimensions of food choices reflect the individual’s cultural and economic environment, and directly influence the eating behavior of workers.

We believe that the results obtained offer a contribution to understand the motivations for the food choices of this population and, therefore, may favor the development of articulated actions for food and nutritional education, using tools that value the dimensions that elicit or influence healthier food choices. For example, the dimensions sensory appeal and health showed strong association with healthier food choices, suggesting a strategy to enhance healthier food habits should consider strengthening those motivations. At the same time, recognizing that people have a tendency to value these dimensions more, this knowledge could be applied in the planning of menus more in line with workers’ consumption expectations.

On the other hand, knowing the positive and negative associations between the influencing factors and the food groups or the processing levels makes it possible to outline strategies that result in the valorization of foods that should be consumed more often, due to the known beneficial role they play (unprocessed food, fruits, whole grains, white meats, among others). Simultaneously, those strategies could discourage the consumption of those known to be more hazardous to health (ultra-processed foods, for example). In the medium and long term, these measures are likely to have a positive effect on the nutritional status of workers.

In addition, public food policies aimed at specific population groups can find in these results a way to offer food groups positively related to the most important dimensions, in such a way that this results in a healthier diet.

This study presents several limitations to generalizability. Although we surveyed an entire federation State, the study was limited to a single geographic location. However, we do not expect to find significant regional differences in the relationship between food choices and energy consumption because the basic nutrition pattern is similar across all Brazilian States and regional foods do not have much expression in everyday diet. The survey was limited to three economically important sectors of activity, but other relevant sectors such as automobile and electronic industries were not surveyed. We used the 24HDR method in the quantification of nutrient consumption that, although being a widely used methodology, it is known to be have reliability issues. On the other hand, we believe that the results have high internal validity due to the coverage of the survey to an entire State, the selection of a large probability sample representative of the target population, the use of a validated questionnaire to evaluate the motivations for food choices, and the nutritional assessment by personal interview with experienced nutritionists.

Therefore, the results obtained in the present study confirmed that among workers, several motivations influence everyday food decisions with different levels of importance, depending on the food categories. Thus, our findings shed some light and provide a preliminary understanding of the relationship of food choice motivations and the consumption of specific foods groups.

Further research in this topic should try to cover workers in other industry sectors and in different geographic and economic areas in order to appraise variations in the motivations for food choices and to improve understanding of the relationship between the motivations and the actual consumption of food items. Better knowledge of these relationships may set the stage to plan and conduct intervention studies to assess the effectiveness of strategies for modification of food habits among workers

## 5. Conclusions

The most important motivations for food choices among manufacturing workers are sensory appeal, price and convenience, while ethical concern, mood and natural content are the least important. Each motivation is associated with a pattern of selective choice of certain food groups and avoidance of others, several of them having been identified in this study. A relationship between certain food choice motivations and NOVA classes was also found, with health motivation associated with increased consumption of unprocessed foods and weight control motivation with decreased consumption of processed culinary ingredients. The knowledge about the motivations behind the consumption of each food group may help nutritionists, health professionals, and educators to model public policy interventions and health promotion actions involving the food choice behaviors.

## Figures and Tables

**Table 1:** Demographic characteristics of the population of manufacturing workers in the State of Rio Grande do Norte, Brazil, 2017–18.

Variable	Point Estimate	95% Confidence Interval
Age (years), mean	38.3	37.2–39.3
Females, %	44.1	40.0–48.4
Males, %	55.8	51.6–60.0
Married/living with partner, %	62.8	57.6–67.8
Income (minimum wages *), mean	1.55	1.35–1.76
Education ≥ high school, %	63.0	57.7–68.0
In-house training, %	19.3	15.5–23.7
BMI (kg/m^2^), mean	27.5	26.9–28.0
Nutritional status, %		
Underweight	1.1	0.4–3.2
Normal weight	32.9	28.1–38.0
Overweight	38.5	33.6–43.6
Obesity I	20.6	16.5–25.4
Obesity II	5.0	3.1–8.1
Obesity III	2.0	0.9–4.4
Total daily intake (kcal), mean	2046	1960–2133

* Minimum wage in Brazil in 2018 was 954 BRL (about 239 EUR).

**Table 2:** Average scores of each dimension of the Food Choices Questionnaire (FCQ) in the population of manufacturing workers of Rio Grande do Norte, Brazil, 2017–18.

FCQ Dimension	*n*	Mean	95% Confidence Interval
Health	918	2.47	2.37–2.36
Mood	917	2.22	2.12–2.33
Convenience	912	2.67	2.57–2.76
Sensory appeal	919	3.71	3.65–3.77
Natural content	918	2.40	2.27–2.52
Price	914	2.90	2.82–2.99
Weight control	910	2.54	2.42–2.66
Familiarity	919	2.61	2.51–2.71
Ethical concern	921	1.56	1.46–1.66

**Table 3:** Description of food groups and food group components consumed by 5% or more of the manufacturing workers of the State of Rio Grande do Norte, Brazil, 2017–18.

Food Groups	Components	Consumers %	% of Total Daily Kcal
White meat	Poultry, turkey, fish, and seafood	68.5	9.42
Breads	White, toasted, sweet and cheese breads	56.0	8.59
Red meat	Beef, pork, ribs, jerked beef, lamb, and meat-based preparations, etc.	57.0	7.77
Rice	White rice and rice-based preparations	82.6	7.30
Milk and dairy	Cheeses, whole, and skimmed milk, butter, dairy drinks, and yogurts	71.8	6.56
Tubers and roots	Potato, yams, sweet potato, cassava, not including industrialized forms	44.2	6.00
Beans	Black, brown, and ‘green’ beans	84.4	5.67
Salt (g)	Added salt	99.8	5.52
Corn	Cornmeal, popcorn, and corn-based preparations	49.2	5.34
Biscuits	Savory and sweet biscuits	36.6	5.10
Pasta	Spaghetti, ravioli, lasagna, and pasta dishes with or without sauce	45.6	4.92
Sugar	Added sugar, white or brown	93.2	4.35
Plant oils	Soy oil	86.8	3.05
Fruits	Fresh fruits and fruit salads	45.1	2.90
Processed meats	Ham, salami, mortadella, sausage, and hamburger	33.5	2.79
Fried and baked snacks	‘Esfiha,’ ‘coxinha,’ ‘empada,’ croissant, pastry, and savory pie	15.7	2.49
Eggs	Omelets, boiled, fried and scrambled eggs	27.3	2.42
Sweets and desserts	Sweets, fruit-based desserts, chocolate, and ice cream	22.5	1.84
Cakes	Homemade cakes	13.4	1.73
Whole grains	Brown rice, oats, pasta, and bread wholemeal	10.3	1.48
Fast foods	Sandwiches, fries, hot dog	5.1	1.30
Soups	Vegetable-based soups and creams	16.2	1.29
Margarine	Margarine or hydrogenated oils	35.0	1.14
Coffee and tea	Coffee, cappuccino, tea, and coffee-based drinks	88.0	1.06
Vegetables	Carrots, tomato, broccoli, cucumber, onion, bell pepper, etc.	88.4	1.05
Fruit juices	Natural fruit juices with or without sugar	54.0	1.00
Sugary drinks	Soft drinks, ready-to-drink juices, powder juice, iced teas	19.4	0.63
Artificial sweeteners (g)	Non-sugar sweeteners	9.1	0.41
Natural spices	Garlic, saffron, chives, oregano, pepper, tomato, onion, and bell pepper	94.5	0.32
Industrialized condiments	Mustard, ketchup, soy sauce, industrialized sauces, etc.	51.8	0.27
Leafy vegetables	Lettuce, cabbage, kale, spinach, Swiss chard etc.	20.1	0.04

The following food groups were consumed by less than 5% of the worker population and were excluded from analysis: legumes, granola (muesli), oilseeds, alcohol, child cereals, snacks, stuffed biscuits, ‘rapadura’ (unrefined sugar blocks), chocolate powder, olive oil, bean stew, dietetic supplements, and tripe/chitterlings.

**Table 4:** Daily food consumption from NOVA four food classes among manufacturing workers of Rio Grande do Norte, Brazil 2017–18.

NOVA Classification	Daily Kcal Consumption		Consumers %
	Daily Kcal	% of Total Daily Kcal	
Unprocessed or minimally processed foods ¹	1094.0	55.6	100
Processed culinary ingredients ^2^	166.5	8.03	100
Processed foods ^3^	289.5	16.1	90.8
Ultra-processed foods ^4^	434.9	20.6	95.4

¹ Includes fresh, chilled, frozen, and dried fruits, vegetables, and roots; natural spices, grains, rice, beans, and corn; red and white meat, eggs, milk, etc. ^2^ Substances used to make hand-made culinary preparations such as salt, oils, and sugar. ^3^ Includes breads, cheeses, salted and cured meats, canned or bottled vegetables, and fruits. ^4^ All foods formulated by industry such as confectionery products, processed meats, fast food, carbonated drinks, and margarine.

**Table 5:** Change in the relative contribution of each food group to total daily energy consumption according to the different motivations for food choice in manufacturing workers of the State of Rio Grande do Norte, Brazil, 2017–18.

FCQ Dimensions	Food Groups	Change in Contribution to Daily Kcal Intake			*p*-Value
		(%)	95% Confidence interval		(Multiplicity adjusted)
Health	Whole grains	0.72	0.45	1.00	<0.001
	Eggs	0.26	0.08	0.44	0.065
	Leafy vegetables	0.16	0.05	0.27	0.053
	Breads	−1.21	−1.97	−0.44	0.030
	Pasta	−1.46	−2.17	−0.74	0.002
Mood	Margarine	−0.16	−0.28	−0.05	0.067
	Salt (g)	−0.31	−0.47	−0.15	0.006
	Pasta	−0.78	−1.22	−0.33	0.011
Convenience	Red meat	1.07	0.34	1.80	0.053
Sensory appeal	Industrialized condiments	0.09	0.03	0.14	0.022
	Eggs	−1.61	−2.50	−0.72	0.009
Natural content	Salt (g)	−0.22	−0.37	−0.06	0.065
	Pasta	−0.84	−1.38	−0.30	0.031
Price	Plant oils	−0.31	−0.50	−0.12	0.022
Weight control	Tubers and roots	1.58	0.61	2.55	0.022
	Whole grains	0.83	0.41	1.24	0.003
	Fruits	0.62	0.40	0.84	<0.001
	Margarine	−0.22	−0.35	−0.10	0.011
	Salt (g)	−0.35	−0.47	−0.23	<0.001
	Breads	−1.16	−1.67	−0.65	<0.001
	Pasta	−1.53	−1.96	−1.10	<0.001
Familiarity	Beans	0.62	0.46	0.78	<0.001
	Soups	0.48	0.23	0.72	0.004
Ethical concern	White meat	1.83	0.53	3.12	0.065
	Pasta	−0.73	−1.01	−0.44	<0.001

## Supplementary Materials

The following are available online at https://www.mdpi.com/2072-6643/12/5/1490/s1, DataFile.xlsx.
